# The inflammatory pathology of dysferlinopathy is distinct from calpainopathy, Becker muscular dystrophy, and inflammatory myopathies

**DOI:** 10.1186/s40478-022-01320-z

**Published:** 2022-02-08

**Authors:** Nicole Becker, Steven A. Moore, Karra A. Jones

**Affiliations:** Department of Pathology, University of Iowa, 200 Hawkins Drive, Iowa City, IA 52242 USA

**Keywords:** Dysferlinopathy, Calpainopathy, Becker muscular dystrophy, Inflammatory myopathy, Complement C5b-9, MHC class I

## Abstract

The descriptions of muscle pathology in dysferlinopathy patients have classically included an inflammatory infiltrate that can mimic inflammatory myopathies. Based on over 20 years of institutional experience in evaluating dystrophic and inflammatory myopathy muscle biopsies at the University of Iowa, we hypothesized the inflammatory histopathology of dysferlinopathy is more similar to limb-girdle pattern muscular dystrophies such as calpainopathy and Becker muscular dystrophy, and distinct from true inflammatory myopathies. Muscle biopsies from 32 dysferlinopathy, 30 calpainopathy, 30 Becker muscular dystrophy, and 30 inflammatory myopathies (15 each of dermatomyositis and inclusion body myositis) were analyzed through digital quantitation of CD3, CD4, CD8, CD20, and PU.1 immunostaining. The expression of MHC class I and deposition of complement C5b-9 was also evaluated. Dysferlinopathy, calpainopathy, and Becker muscular dystrophy muscle biopsies had similar numbers of inflammatory cell infiltrates and significantly fewer CD3+ T-lymphocytes than dermatomyositis (p = 0.05) and inclusion body myositis (p < 0.0001) biopsies. There was no statistically significant difference in the number of PU.1+ macrophages identified in any diagnostic group. MHC class I expression was significantly lower in the limb-girdle pattern muscular dystrophies compared to the inflammatory myopathies (p < 0.0001). In contrast, complement C5b-9 deposition was similar among dysferlinopathy, dermatomyositis, and inclusion body myositis biopsies but significantly greater than calpainopathy and Becker muscular dystrophy biopsies (p = 0.05). Compared to calpainopathy, Becker muscular dystrophy, and inflammatory myopathies, the unique profile of minimal inflammatory cell infiltrates, absent to focal MHC class I, and diffuse myofiber complement C5b-9 deposition is the pathologic signature of dysferlinopathy muscle biopsies.

**Supplementary Information:**

The online version contains supplementary material available at 10.1186/s40478-022-01320-z.

## Introduction

Dysferlinopathy, now classified as limb-girdle muscular dystrophy R2, is an autosomal recessive limb-girdle muscular dystrophy (LGMD) caused by pathogenic variants in the *DYSF* gene located on chromosome 2p13 that encodes the protein dysferlin [2, 3]. Dysferlin is a calcium ion sensor located in the sarcolemma of myofibers; pathogenic variants in *DYSF* lead to defective repair of the sarcolemma resulting in muscle degeneration [2]. Clinically, patients may present with a distal phenotype of muscle weakness, known as Miyoshi myopathy, or more often, a proximal phenotype, historically known as LGMD 2B. Regardless of the phenotype, the muscle pathology is indistinguishable and includes complete or nearly complete loss of dysferlin expression, especially at the sarcolemma [1, 22, 23].

Classically, the muscle pathology of dysferlinopathy biopsies is described to include inflammatory cell infiltrates mimicking inflammatory myopathies [7, 8, 11, 17, 26]. Dysferlinopathy biopsies also demonstrate active myonecrosis and regeneration like other muscular dystrophies. In contrast, multiple other limb-girdle pattern muscular dystrophies, including those with pathogenic variants in *calpain-3* (*CAPN3*), *dystrophin* (*DMD*), and *fukutin-related protein* (*FKRP*) may have inflammatory cell infiltrates in muscle biopsy specimens yet they are typically not confused with inflammatory myopathies [13, 15].

Prior studies have characterized the inflammatory cell components in small numbers of dysferlinopathy muscle biopsies often compared to diagnoses such as polymyositis, dermatomyositis, and Duchenne muscular dystrophy (DMD) [4, 6–8, 11, 26]. Due to the presumptive inflammatory nature of dysferlinopathy biopsies, MHC class I (HLA ABC) and complement C5b-9 (MAC) deposition have also variably been evaluated and reported [4–8, 11, 26]. In these previous studies, dysferlinopathy cases were most commonly diagnosed based on a lack of dysferlin protein expression by immunostaining or Western blot analysis with only case reports or small series identifying cases by the presence of definitive pathogenic variants in *DYSF* [4–8, 11, 17, 26].

Despite this extensive literature, the experience of providing diagnostic services at our institution has suggested the inflammatory pattern of dysferlinopathies is distinct from true inflammatory myopathies and more similar to other limb-girdle pattern muscular dystrophies. Therefore, we set out to assess the distribution, type, and number of inflammatory cells in dysferlinopathy, calpainopathy, and Becker muscular dystrophy cases compared to two common inflammatory myopathies: dermatomyositis and inclusion body myositis. Inflammatory cell quantitation was accomplished with the assistance of digital image analysis. In addition, we sought to better characterize the expression of MHC class I and deposition of complement C5b-9 in dysferlinopathy compared to the other diagnoses.

## Materials and methods

### Case selection

This retrospective study utilizing archival tissue was approved by the University of Iowa Institutional Review Board. Cases of dysferlinopathy (DYSF), Becker muscular dystrophy (BMD), calpainopathy (CAPN3), dermatomyositis (DM), inclusion body myositis (IBM), and normal controls were identified through a retrospective review of muscle biopsies diagnostically evaluated in the University of Iowa Department of Pathology between 1997 and 2019. The DM and IBM cases were consecutively chosen cases from the years 2017 to 2019. All file copy H&E-stained slides were reviewed for the histologic quality of the biopsies. To be selected for the study, each case was required to have stored frozen tissue sufficient for the testing detailed below. Thirty-two DYSF cases were selected with confirmed genetic pathogenic variants in the *DYSF* gene, negative or greatly reduced dysferlin protein expression in Western blot analysis, and/or loss or greatly reduced immunofluorescence staining for dysferlin. Dysferlin evaluation was performed as previously published [18]. Anti-dysferlin monoclonal antibodies utilized were Hamlet and Hamlet-2 (Leica Biosystems; Buffalo Grove, IL), and Romeo (Abcam; Waltham, MA). Thirty BMD cases and 30 CAPN3 cases were selected with abnormal protein expression by immunostaining or Western blot analysis and/or confirmed genetic pathogenic variants in the *DMD* or *CAPN3* genes, respectively. Dystrophin and calpain-3 protein evaluation was performed as previously published [20]. Anti-dystrophin antibodies included a polyclonal carboxy terminus antibody (Abcam; ab15277) and monoclonal antibodies directed at epitopes in exons 1, 7/8, 10–12, 20/21, 27, 31/32, 38/39, 43, 45, 46, 47, 47/48, 48–50, and 50 (Developmental Studies Hybridoma Bank; The University of Iowa). Anti-calpain-3 antibodies were 12A2 and 2C4 (Leica Biosystems). For those biopsies that did not previously have genetic testing confirmation, DNA was isolated from frozen tissue and analyzed by next-generation sequencing of *CAPN3, DMD,* or *DYSF* (Prevention Genetics; Marshfield, WI). A total of 30 inflammatory myopathy cases (15 DM and 15 IBM cases) were chosen for comparison. Thirty muscle biopsies without diagnostic histopathologic abnormalities across an age spectrum similar to the muscular dystrophy cases were selected for analysis as normal controls.

### Biopsy sectioning and staining

All cases had tissue previously frozen and stored at − 80 °C. Frozen tissue was recut at a thickness of 10 microns for H&E staining, immunohistochemistry, and immunofluorescence staining. Cryosections were stained using DAKO immunohistochemical staining instrumentation for CD3 (Agilent (DAKO); Santa Clara, CA; polyclonal; 1:800), CD4 (Leica Biosystems; clone 4B12; 1:100), CD8 (Agilent (DAKO); clone C8/144B; 1:200), CD20 (Agilent (DAKO); clone L26; 1:1600), and PU.1 (BD Pharmingen; San Diego, CA; clone G148-74; 1:100). Immunofluorescence staining was performed using HLA-ABC (MHC class I; Agilent (DAKO); clone W6/32; 1:25) and complement C5b-9 (Abcam; clone aE11; ab66768; 1:50). All H&E and immunostained slides were digitally scanned at 20× magnification using the 3DHistech PANNORAMIC 1000 slide scanner (Budapest, Hungary) and then evaluated using the companion CaseViewer and QuantCenter applications (3DHistech).

### Western blotting

Western blot analysis was performed on 24 DYSF cases and all CAPN3 cases using pooled cryosections. The cryosections were homogenized followed by protein separation on a 3–13% gradient gel. The antibodies used for dysferlin protein expression were Hamlet (1:500) and Hamlet-2 (1:1000). The antibodies used for calpain-3 protein expression were 12A2 (1:50) and 2C4 (1:25). All antibodies were purchased from Leica Biosystems. Results were expressed as absent or reduced for DYSF cases and absent, reduced, or nearly normal with abnormal degradation products for CAPN3 cases.

### Inflammatory cluster counting

Clusters of inflammatory cells were counted in one H&E section from each muscle biopsy. An inflammatory cluster was defined as a group of ≥ 20 inflammatory cells not associated with myonecrosis or regeneration. Sites of myonecrosis and regeneration were excluded from the cluster counting because the process of necrosis/regeneration elicits a local, mixed inflammatory cell response independent of the etiology. Our goal was to identify and quantify perimysial or endomysial chronic inflammatory cell clusters more directly related to disease etiology. Inflammatory cluster counts were performed independently by three pathologists. Cases with discrepant cluster counts were reviewed concurrently by all three pathologists and a consensus count was determined. Cluster counts were normalized using the cross-sectional area of each biopsy section, which was determined using the CaseViewer software from the digital whole slide image.

### Immunohistochemistry digital image analysis

Immunohistochemical staining for inflammatory cells was performed on all cases, including normal controls. The number of immunopositive inflammatory cells in each biopsy was counted using the QuantCenter application of the CaseViewer program. Protocols for each immunohistochemical stain were individually optimized prior to the digital image analysis counting. Cells within each biopsy were digitally counted as positive (3+ intensity) or negative (0). The number of positive cells in each case for each immunohistochemical marker was normalized over the cross-sectional area, which was determined using the CaseViewer software from the digital whole slide image.

### Immunofluorescence analysis

MHC class I and complement C5b-9 were performed on all DYSF, BMD, CAPN3, DM, and IBM cases. Negative/normal control tissue was stained on the same slides as the experimental sections. Sarcolemmal staining with either antibody was scored as negative, focal, or diffuse relative to the entire biopsy cryosection.

### Statistical analysis

The mean, standard deviation, and standard error were calculated for inflammatory cluster counts and each immunohistochemical stain. Statistical significance was defined as p < 0.05. Comparison of subject sex and age for each diagnosis was performed and subsequently used as covariates in the One-way ANOVA model that tests for diagnosis effect and the post-hoc Tukey–Kramer test for pairwise comparisons of diagnosis group means. Data for each immunohistochemical stain were skewed to the right. Therefore, the natural log transformation, Ln(Y), was applied to normalize the data distribution for statistical analysis. The 95% confidence intervals and p-value of pairwise comparisons were adjusted using Tukey–Kramer method to account for multiplicity in the analyses for immunostaining. Inflammatory clusters were compared among diagnosis groups using Kruskal–Wallis test, with pairwise comparisons using Dwass, Steel, Critchlow-Fligner (DSCF) multiple comparison analysis. Immunofluorescence results were compared using Pearson Chi-square exact test, with p-values for pairwise comparisons adjusted using Bonferroni’s method.

## Results

### Demographics

Thirty-two patients with pathogenic variants in the *DYSF* gene were identified, including 24 males. Of the 32 patients, 31 had pathogenic variants identified in both alleles. One case had only one pathogenic variant identified; however, no dysferlin was identified in this muscle biopsy by immunofluorescence staining or by Western blotting. A supplemental table lists the pathogenic variants for each dysferlinopathy case (Additional file 1). The age at time of biopsy ranged from 13 to 70 years. The muscle biopsies were determined to have no dysferlin or greatly reduced dysferlin by immunostaining and western blotting (Additional file 1). Thirty cases each of BMD, CAPN3, inflammatory myopathy, and normal controls were chosen for comparison to the DYSF cases. Pathogenic *DMD* variants were identified in 27 BMD cases. The other 3 BMD cases included one with a variant of uncertain significance (VUS) and two without variants identified in the *DMD* gene despite abnormal immunostaining for dystrophin. The possibility of *DMD* variants in non-coding regions of the gene was not pursued for this study. At least one pathogenic *CAPN3* variant was identified in all but one of the CAPN3 cases. Ten cases had one pathogenic variant and one normal allele, including 3 cases with known dominant mutations. One case had no pathogenic variants or VUS identified even though no calpain-3 was identified by Western blotting. The possibility of *CAPN3* variants in non-coding regions of the gene was not pursued for this study. Dystrophin expression was evaluated by immunostaining in all BMD cases; calpain-3 expression was evaluated by Western blotting in all CAPN3 cases (Additional file 1). The demographics for each case including sex distribution and age range at the time of biopsy are summarized in Table 1.

**Table 1:** Diagnoses with sex and age demographics at time of muscle biopsy

Diagnosis (abbreviation/gene)	Number of cases	Sex (M:F)	Median age at biopsy (range)
Dysferlinopathy (*DYSF)*	32	24:8	27.5 years (14–68)
Becker muscular dystrophy (BMD/*DMD*)	30	30:0	14 years (4–84)
Calpainopathy (*CAPN3)*	30	16:14	28.5 years (5–71)
Inflammatory myopathy	30	13:17	63 years (3–84)
Dermatomyositis (DM)	15	1:14	34 years (3–72)
Inclusion body myositis (IBM)	15	12:3	68 years (51–84)
Normal controls	30	11:19	34 years (12–74)

### Inflammatory cluster counting

**Fig. 1:**
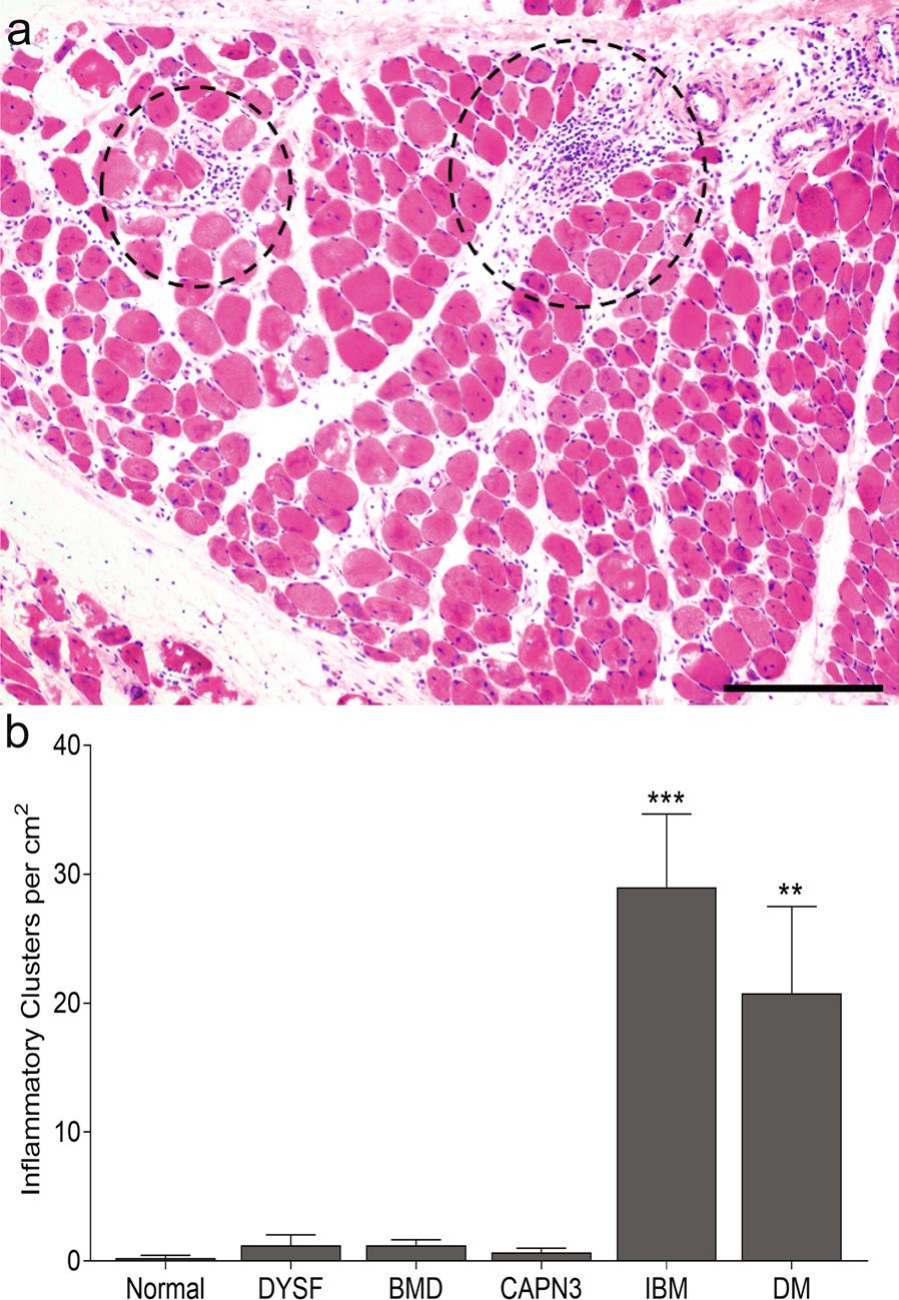
Inflammatory cluster counting. **a** Representative inflammatory clusters (dashed line circles) of ≥ 20 inflammatory cells not associated with myonecrosis within the perimysial (upper right) and endomysial (upper left) space of the muscle biopsy. H&E staining; size bar is 200 µm. **b** Inflammatory clusters per diagnosis. Data expressed as mean ± SEM. **p ≤ 0.002. ***p < 0.0001. *DYSF* dysferlinopathy, *BMD* Becker muscular dystrophy, *CAPN3* calpainopathy, *DM* dermatomyositis, *IBM* inclusion body myositis

Inflammatory cluster counting in the dystrophy cases identified a similar mean number of clusters normalized by cross-sectional area. The range of inflammatory clusters in the DYSF cases was 0 to 25 clusters per cm^2^ (mean 1.2 clusters per cm^2^). Only one DYSF case had > 10 clusters per cm^2^. For BMD and CAPN3 cases the range per cm^2^ was 0 to 7.7 clusters (mean 1.2 clusters per cm^2^) and 0 to 8.2 clusters (mean 0.7 clusters per cm^2^), respectively. There were significantly more clusters in each of the inflammatory myopathy categories. DM cases ranged 0 to 86.6 clusters per cm^2^ with a mean of 20.8 clusters per cm^2^ (p = 0.002 compared to BMD cases; p ≤ 0.0001 compared to DYSF, CAPN3, and control cases). IBM cases ranged from 3.8 to 77.8 clusters per cm^2^ with a mean of 29 clusters per cm^2^ (p ≤ 0.0001 compared to DYSF, BMD, CAPN3, and control cases). There was no statistically significant difference in the number of clusters between DM and IBM cases. See Fig. 1a for a representative image of inflammatory clusters and Fig. 1b for a comparison of inflammatory clusters by diagnosis. Inflammatory clusters were seen in both an endomysial and perimysial distribution. The normal controls had one biopsy with a single perimysial inflammatory cluster; otherwise, no inflammatory clusters were identified in the control group.

### Immunohistochemistry quantitation

**Fig. 2:**
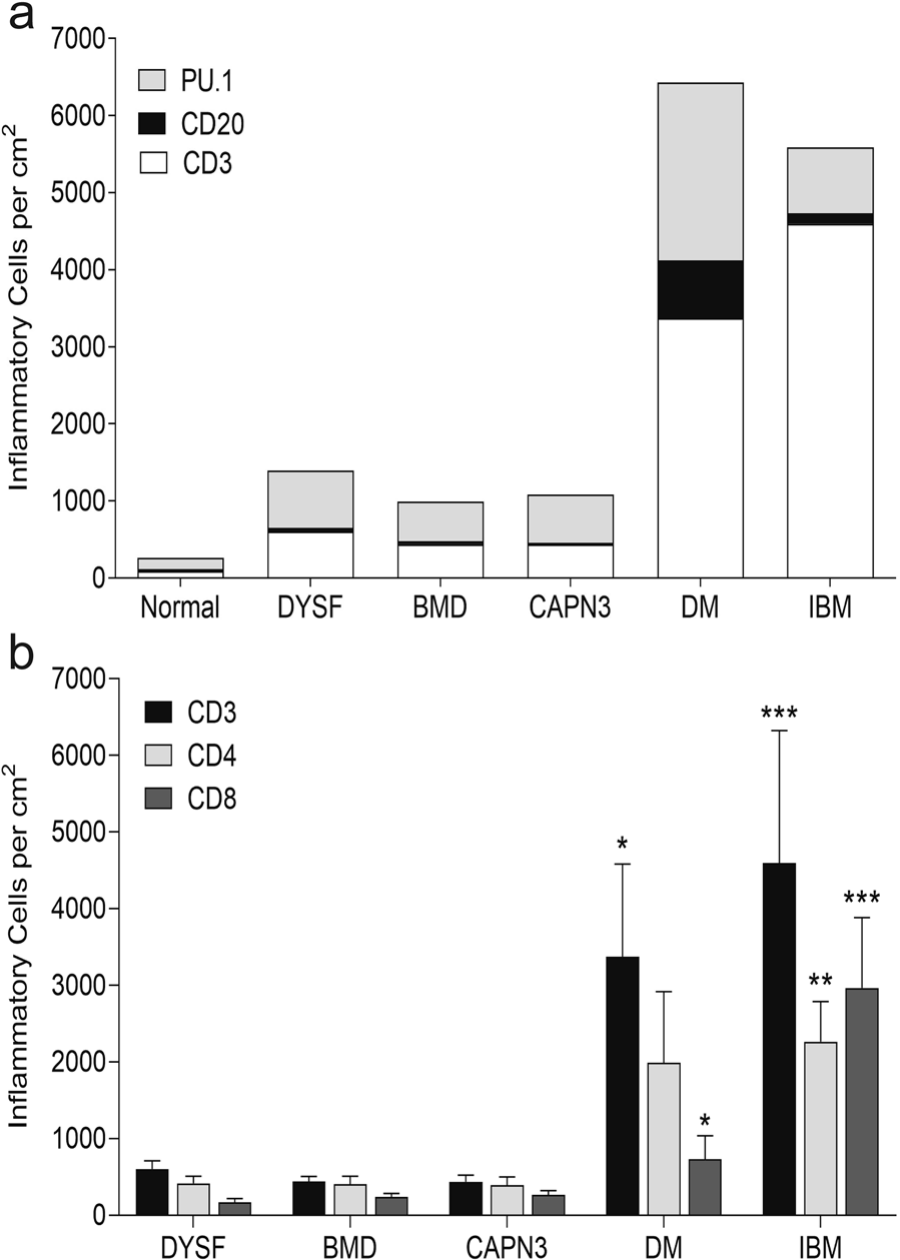
Inflammatory infiltrates in dystrophy and inflammatory myopathy cases. **a** The combined number of CD3, CD20, and PU.1 inflammatory cells for each diagnosis. All data expressed as the mean per cm^2^. **b** The number of CD3, CD4, and CD8 cells for each diagnosis. All data expressed as the mean ± SEM. *p = 0.05. **p = 0.01. ***p < 0.0001. *DYSF* dysferlinopathy, *BMD* Becker muscular dystrophy, *CAPN3* calpainopathy, *DM* dermatomyositis, *IBM* inclusion body myositis

**Fig. 3:**
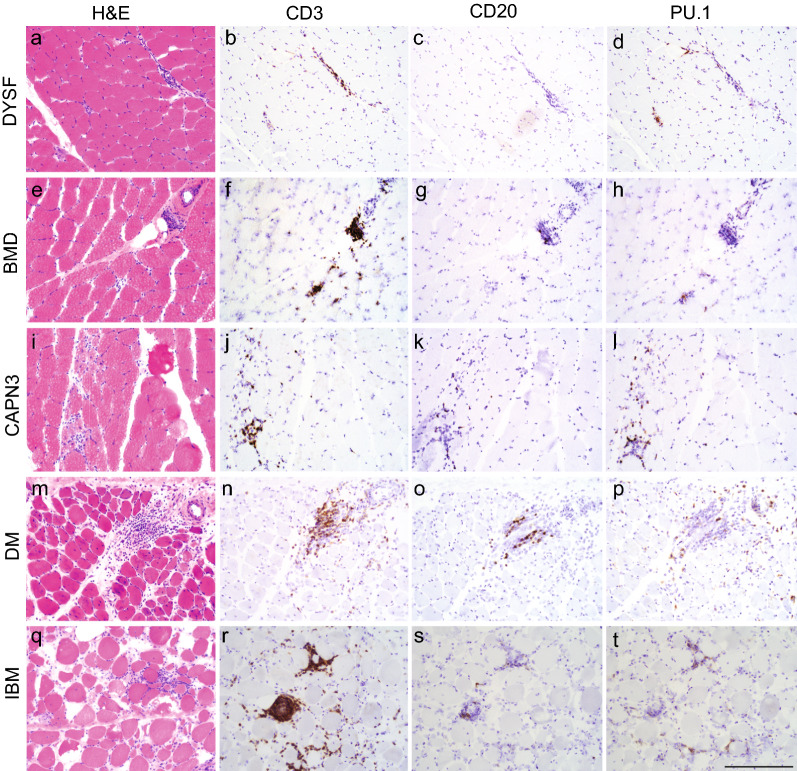
Representative images of dystrophy and inflammatory myopathy inflammatory infiltrates. Histologic and immunohistochemical findings of the cellular infiltrates in DYSF (**a**–**d**), BMD (**e**–**h**), CAPN3 (**i**–**l**), DM (**m**–**p**), and IBM (**q**–**t**). H&E and immunohistochemical staining for CD3, CD20, and PU.1; size bar in panel t is 200 µm and equivalent for all photomicrographs. *DYSF* dysferlinopathy, *BMD* Becker muscular dystrophy, *CAPN3* calpainopathy, *DM* dermatomyositis, *IBM* inclusion body myositis

Digital quantitation of the number of inflammatory cells per cm^2^ revealed more inflammatory cells in both subtypes of inflammatory myopathy compared to the dystrophy cases (Fig. 2 and Additional file 2). There were significantly more CD3+ cells in the IBM cases (p < 0.0001) and DM cases (p = 0.05) compared to all dystrophy and control cases. There was no statistically significant difference in the numbers of CD3+ cells in each of the dystrophy diagnoses compared to each other. IBM cases had significantly more CD4+ cells compared to all dystrophy cases and controls (p ≤ 0.003) and significantly more CD8+ cells compared to all other diagnoses including dermatomyositis cases (p ≤ 0.006). In contrast, the DM cases had significantly more CD8+ cells compared to the DYSF cases (p = 0.05), but otherwise there were no significant differences in the number of CD4+ and CD8+ cells. CD20+ cells were the least numerous cell type in all diagnoses. The inflammatory myopathies had significantly higher numbers of CD20+ cells compared to controls (p = 0.05), and DM cases had significantly more CD20+ cells compared to normal controls (p = 0.002), BMD (p = 0.013) and CAPN3 (p = 0.005) cases. DM trended towards more CD20+ cells compared to DYSF but was not statistically significant (p = 0.052). The most prevalent cell type in the dystrophy cases was PU.1+ cells. PU.1 is a transcription factor that is expressed strongly in macrophages and at a low to moderate level in B-lymphocytes [14, 21]. As the number of CD20+ B-lymphocytes in all biopsies examined was very low, it is likely the vast majority of PU.1+ cells were macrophages in all diagnoses. There were significantly more PU.1+ cells in all myopathy cases compared with the controls but otherwise there were no significant differences between the numbers of PU.1+ cells in any of the dystrophies or inflammatory myopathy cases. Figure 3 demonstrates a representative staining pattern of inflammatory cells by diagnosis.

### Immunofluorescence staining pattern

**Fig. 4:**
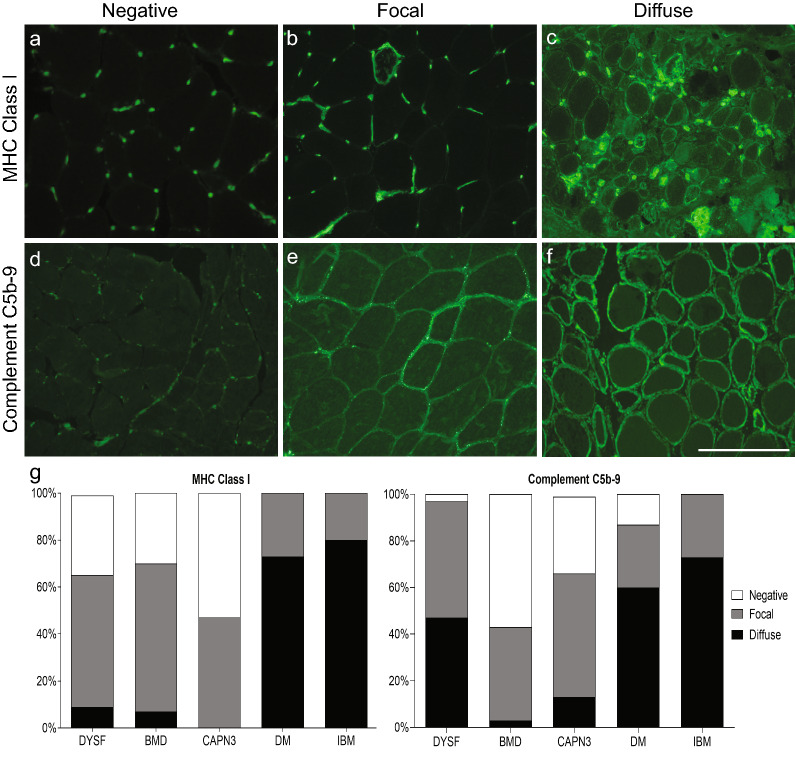
MHC class I and complement C5b-9 immunofluorescence staining in dystrophies and inflammatory myopathies. Most DYSF cases had negative (**a**) or focal (**b**) MHC class I expression. Diffuse (**c**) MHC class I expression was seen in only 9% of cases. For complement C5b-9 deposition, 3% of DYSF cases were negative (**d**), while 50% demonstrated focal (**e**) and 47% had diffuse (**f**) deposition. The percentage of negative, focal, and diffuse MHC class I expression and complement C5b-9 deposition in each diagnosis (**g**). The size bar in panel f is 200 µm and equivalent for all photomicrographs. *DYSF* dysferlinopathy, *BMD* Becker muscular dystrophy, *CAPN3* calpainopathy, *DM* dermatomyositis, *IBM* inclusion body myositis

The pattern of MHC class I expression was very similar in the majority of the dystrophy cases and reported as either negative or focal. Three DSYF cases (9%) and 2 BMD cases (7%) demonstrated diffuse MHC class I expression (Fig. 4c and g). No CAPN3 cases had diffuse MHC class I expression. There was no statistical difference in the staining pattern among the dystrophies. In comparison, all DM and IBM cases demonstrated diffuse or focal (perifascicular in the setting of DM) staining which was statistically different from the dystrophies (p < 0.001). In contrast to MHC class I staining, DYSF cases had significantly more complement C5b-9 deposition on the sarcolemma of non-necrotic muscle fibers compared to BMD and CAPN3 cases (p = 0.05) (Fig. 4f and g). There was no significant difference between complement C5b-9 deposition in inflammatory myopathy and DYSF cases.

## Discussion

The aim of the present study was to determine if the skeletal muscle in dysferlinopathy has a unique profile of inflammatory cell infiltrates or inflammatory marker expression. By evaluating the largest cohort of genetically confirmed dysferlinopathy cases reported to date and comparing them to similar numbers of Becker muscular dystrophy, calpainopathy, and inflammatory myopathy cases, this study demonstrates that the number of inflammatory cell clusters and total number of inflammatory cells are similar in the muscle biopsies from DYSF, CAPN3, and BMD patients. Each of these dystrophy cases have fewer inflammatory cells than DM and IBM cases (Figs. 1 and 2). This indicates that the degree and pattern of cellular inflammation in inflammatory myopathies is substantially different from the dystrophies, including dysferlinopathy, supporting our initial hypothesis.

The inflammatory cluster counting was a unique aspect to this study and highlights a method to distinguish muscular dystrophies from inflammatory myopathies. Numerous previous studies have evaluated the distribution of inflammatory cells in DYSF biopsies [4–7, 11, 17]. McNally et al. noted two of the four biopsies evaluated in their study had perivascular inflammatory infiltrates but all four of their biopsies had a necrotizing myopathy [17]. In addition, Brunn et al. and Cenacchi et al. commented on the associated myonecrosis present in the inflammatory infiltrates of the majority of their biopsies [4, 5]. Two other studies that had previously evaluated the inflammatory components of DYSF muscle biopsies described myogenic and dystrophic changes rather than inflammatory cell infiltrates [8, 19]. This study confirms that DYSF biopsies and other muscular dystrophy biopsies lack significantly increased inflammatory cell infiltrates that are independent of the physiologic processes of myonecrosis and regeneration. Of note, a single DYSF biopsy did contain > 10 inflammatory clusters/cm^2^; however, this tissue lacked MHC Class I expression in contrast to inflammatory myopathy biopsies with a similar number of inflammatory clusters. Therefore, it is important to distinguish patterns of myonecrosis and inflammatory cell infiltrates when considering a differential diagnosis of a dystrophy and myositis.

Another unique aspect to this study was the use of digital image analysis when counting inflammatory cells. Prior studies that quantitated inflammatory cells in DYSF and inflammatory myopathy biopsies either gave visual impressions of the biopsies, counted limited numbers of random microscopic fields, or counted hot spot areas in each biopsy [4, 6–8, 11, 26]. This limits the overall evaluation and impression of the biopsy. The current study utilized whole slide imaging and automated quantitation techniques to evaluate the entire biopsy in each case and give a more accurate overall number of cells present in each of the muscular dystrophy and inflammatory myopathy biopsies.

In addition to a difference in the overall number of inflammatory cells between muscular dystrophy and inflammatory myopathy biopsies, the type of inflammatory cells present is also distinct. In the DYSF biopsies, like the CAPN3 and BMD biopsies, PU.1+ cells (predominantly macrophages) were most prevalent. The number of PU.1+ cells was not significantly different from the inflammatory myopathy biopsies, but the number of CD3+ lymphocytes was significantly lower in the dystrophy cases. The presence of increased numbers of macrophages in DYSF biopsies has been observed in other studies and is not surprising due to the presence of myonecrosis [4–7, 11, 26]. Many of these studies also noted the increased number of CD4+ cells but did not distinguish between T-lymphocytes and macrophages [4–6, 26]. It is known that both T-lymphocytes and macrophages can show immunoreactivity for CD4. Gallardo et al. did note the majority of CD4+ cells in their biopsies were actually macrophages due to concomitant expression of CD68 [11].

Through the comparison of CD4+ cells with the CD3+ T-lymphocytes and PU.1+ macrophages, the current study confirms that the CD4+ cells present in DYSF biopsies are a mixture of CD4+ T-lymphocytes and macrophages. We also demonstrated that the quantity of these inflammatory infiltrates is similar in number to CAPN3 and BMD muscles biopsies. Confalonieri et al. and Yin et al. previously compared the inflammatory cells of DYSF and Duchenne muscular dystrophy biopsies. Confalonieri et al. described a similar amount of inflammation between DYSF and DMD biopsies [7]. In contrast, Yin et al. documented the presence of more macrophages in DYSF biopsies compared to DMD biopsies [26]. In contrast to prior studies, the current study is the first to report on the inflammatory cells in a large cohort of BMD or CAPN3 biopsies compared to DYSF cases, as all these diagnoses more closely mimic each other in the severity of dystrophy. This study demonstrates a similar number of T-lymphocytes, B-lymphocytes, and macrophages in all three of these diagnoses.

In comparison to the muscular dystrophy biopsies, the myositis cases had significantly more total inflammatory cells with the most prevalent cell type in both DM and IBM being CD3+ T-lymphocytes. The most common inflammatory myopathy diagnosis used in previous studies to compare with DYSF was polymyositis [4, 6, 7, 11, 26]. Because the diagnostic term of polymyositis is becoming obsolete with current autoantibody testing allowing for a more specific classification of inflammatory myopathies (eg, anti-tRNA synthetase syndrome-related myositis), DM and IBM cases were chosen for the present study. Two previous studies used DM and/or IBM in their comparisons [4, 6]. In the study by Brunn et al., one case of DM and one case of IBM were compared with DYSF biopsies and both inflammatory myopathies showed more CD4+ and CD8+ cells [4]. Choi et al. compared four DYSF biopsies with four cases of DM and noted a similar proportion of macrophages in these two groups [6]. Both of these studies evaluated far fewer cases compared to the present study, but the presence of a similar proportion of macrophages in DM and IBM biopsies compared to DYSF biopsies was similar. In contrast, neither of the prior studies discussed the use of CD3 to evaluate the number of T-lymphocytes present in the biopsies, which was the most prevalent cell type in biopsies from this study. Therefore, another way to distinguish dystrophy biopsies from inflammatory myopathies is the presence of relatively more macrophages in dystrophies along with absolutely and relatively more lymphocytes in inflammatory myopathies.

In the present study, the combined pattern of immunofluorescence staining for MHC class I and complement C5b-9 was unique in DYSF biopsies compared to CAPN3, BMD, DM, and IBM biopsies. We demonstrated that the expression of MHC class I at the sarcolemma and/or sarcoplasm in DYSF biopsies was most likely to be focal or negative similar to the CAPN3 and BMD biopsies. The vast majority of the inflammatory myopathy biopsies had diffuse or perifascicular staining for both MHC class I and complement C5b-9. Multiple previous studies have documented the expression of MHC class I at the sarcolemma of DYSF biopsies [4–8, 11, 26], however, the localization of MHC class I in these cases was most common in necrotic and regenerating fibers in association with macrophages [5, 7, 8, 11]. Only the study by McNally et al. demonstrated no MHC class I expression in any of the cases analyzed, but this may be due to the small number of biopsies studied [17].

In contrast, complement C5b-9 staining in DYSF cases was almost always present (focal or diffuse) on the surface of muscle fibers, with almost half of the cases having diffuse expression. This distinguishes it from CAPN3 and BMD cases, the majority of which were negative or focal. Complement C5b-9 has been reported to be deposited on the sarcolemma of non-necrotic and cytoplasm of necrotic fibers in dysferlin-deficient mice and in patients with dysferlinopathy, making it distinct from DMD where complement C5b-9 was exclusively identified in the cytoplasm of necrotic fibers [4, 6, 7, 12, 26]. Complement C5b-9 may also distinguish DYSF from other LGMD subtypes beyond those evaluated in this study. An analysis by Kölbel et al. analyzing the inflammatory infiltrates of *FKRP* dystroglycanopathy (LGMDR9) did not identify complement C5b-9 deposition as a component of the muscle pathology despite inflammation associated with regenerating myofibers [15]. This matches the diagnostic experience in dozens of dystroglycanopathy cases over the past 20 years at Iowa (data not shown).

Finally, genetic testing is a highly important aspect of the workup for muscular dystrophies and demonstrates its importance when interpreted in conjunction with muscle biopsy findings. The genetic testing done for the present study produced some interesting findings, including a few variants of uncertain significance (VUS). First, all but one DYSF case was found to have pathogenic variants in both *DYSF* alleles, consistent with the known autosomal recessive inheritance pattern of the disease. One DYSF case was heterozygous for a single pathogenic variant, c.4253G>A, p.G1418D (Additional file 1). This case had a chronic necrotizing myopathy histomorphology with diffuse complement C5b-9 deposition and was negative for dysferlin protein expression by immunofluorescence staining and Western blotting. The pathogenic variant identified was not present in any other DYSF case in our cohort, but has been previously reported to cause LGMD in the homozygous state [24]. Five VUS were identified in 5 separate cases which were determined to be likely pathogenic based on muscle biopsy findings. One VUS was identified in a BMD case (c.239C>A, p.A80E) and four were identified in CAPN3 cases (c.181T>C, p.F61L; c.1063C>T, p.R355W; c.1746-20C>G (splice site); c.2462C>T, p.A821V) (Additional file 1). In the BMD case, immunofluorescence staining supported a deletion of a portion of the dystrophin protein. For the CAPN3 cases, all were heterozygous for their respective VUS and three had a pathogenic variant in the other allele. In the cases with two variants identified, one (c.1746-20C>G) completely lacked calpain-3 protein expression, one (c.181T>C, p.F61L) had a 75% reduction of protein expression, and one (c.1063C>T, p.R355W) had normal protein expression but lacked degradation products by Western blotting. Normal protein expression with a lack of degradation products has been previously described as an expression pattern in a subset of calpainopathy cases [9, 10]. There was no pathogenic variant in the other allele in the fourth case (c.2462C>T, p.A821V). However, the biopsy in this case completely lacked calpain-3 protein expression by Western blotting. This suggests the possibility of an autosomal dominant form of calpainopathy or a not yet identified, recessive second variant. Finally, three of the CAPN3 cases in our cohort were found to be heterozygous for the pathogenic *CAPN3* variant that has been reported in autosomal dominant calpainopathy (LGMD D4) (Additional file 1) [16, 25].

A limitation to the present study was not including immune-mediated necrotizing myopathy (IMNM); a recently recognized diagnosis that may be challenging to distinguish from a muscular dystrophy in muscle biopsies. The pathologic features of immune-mediated necrotizing myopathy have overlap with the biopsy findings in DYSF. These include scattered necrotic and regenerating myofibers with infiltrating macrophages and minimal associated lymphocytic infiltrates. There is often scattered MHC class I expression and complement C5b-9 deposition on the sarcolemma. The ability to distinguish these entities may rely on dysferlin analysis by immunofluorescence, Western blotting, IMNM-related autoantibody testing, and genetic testing. Future work is planned to address the diagnostic differences between dysferlinopathy and IMNM biopsies. An additional limitation is that other forms of myositis including anti-tRNA synthetase syndrome-related myositis and mixed connective tissue disease overlap myositis were not included in this study.

In conclusion, the present study demonstrated a distinct inflammatory pattern in dysferlinopathy biopsies. The cellular infiltrate is similar to other dystrophies with significantly fewer inflammatory cell clusters than inflammatory myopathies. Total lymphocytes in the biopsies of DYSF, CAPN3, and BMD are also quite similar and substantially fewer than DM and IBM. Like BMD and CAPN3, the expression of MHC class I in DYSF is more likely to be focal or negative compared to DM and IBM. In contrast, the greater degree of complement C5b-9 deposition in DYSF distinguishes it from other dystrophies. Therefore, the combined lower number of cellular inflammatory infiltrates with a predominance of macrophages and minimal MHC class I expression, yet substantial deposition of complement C5b-9 is the unique inflammatory pattern of dysferlinopathies.

## Supplementary Information

**Additional file 1**. Genetic testing, Western blot, and immunofluorescence results by diagnosis.

**Additional file 2**. Digital quantitation of inflammatory cells per cm2 by diagnosis.

## Data Availability

All data generated or analyzed during this study are included in this published article and its additional files.

## Abbreviations

BMD: Becker muscular dystrophy
CAPN3: Calpainopathy
DM: Dermatomyositis
DMD: Duchenne muscular dystrophy
DYSF: Dysferlinopathy
IBM: Inclusion body myositis
LGMD: Limb-girdle muscular dystrophy
MAC: Membrane attack complex
MHC: Major histocompatibility complex
VUS: Variant of uncertain significance
