# Supplementary material for: The inflammatory pathology of dysferlinopathy is distinct from calpainopathy, Becker muscular dystrophy, and inflammatory myopathies
Source: Acta Neuropathol Commun. 2022 Feb 8;10:17. doi: 10.1186/s40478-022-01320-z (PMC8822795; doi:10.1186/s40478-022-01320-z)
Supplement: Supplementary file 1 — Additional file 1. Genetic testing, Western blot, and immunofluorescence results by diagnosis. [file 40478_2022_1320_MOESM1_ESM.pdf]

Additional file 1a. Genetic testing results for dysferlinopathy cases

Age (years)	Sex (M/F)	*DYSF* variants		Western blot result	IF[1] staining result
		Allele 1	Allele 2		
14	M	c.2643+1G>A (splice site)	c.1948delC p.L650Yfs*6	0	0
15	M	deletion of exons 3-4	deletion of exons 3-4	0	0
15	M	c.2643+1G>A (splice site)	c.865T>C p.S289P	++	++
17	M	c.3444T>A p.Y1148*	c.4497delT p.F1499Lfs*4	0	0
20	M	c.5979dupA p.E1994Rfs*3	c.5979dupA p.E1994Rfs*3	0	0
20	M	c.3444T>A p.Y1148*	c.5713C>T p.R1905*	0	0
21	M	c.5077C>T p.R1693W	c.5698_5699delAG p.S1900Efs*14	0	0
21	M	c.4253G>A p.G1418D	None identified	0	0
22	F	c.5429G>A p.R1810K	c.5429G>A p.R1810K	0	0
22	M	c.2779del p.A927Lfs*21	c.3478C>T p.Q1160*	0	0
23	M	c.5105T>C p.L1702P	c.5105T>C p.L1702P	ND	0
23	M	c.855+1delG (splice site)	c.3036G>A p.W1012*	0	0
25	F	c.5022delT p.F1674Lfs*48	c.5022delT p.F1674Lfs*48	+	0
26	F	c.2643+1G>A (splice site)	c.2643+1G>A (splice site)	ND	0
27	M	c.755C>T p.T252M	c.5444G>T p.C1815F	0	0

27	M	c.2372C>G p.P791R	c.2372C>G p.P791R	0	0
28	M	c.895G>T p.G299W	c.895G>T p.G299W	ND	0
30	M	c.3167G>A p.R1058Q	c.3167G>A p.R1058Q	0	0
31	M	c.755C>T p.T252M	c.4685dupT p.M1562Ifs*39	ND	0
32	F	c.4081T>C p.C1361R	deletion of exons 25-29	0	0
32	F	c.2643+1G>A (splice site)	c.2643+1G>A (splice site)	0	0
33	M	c.1368C>A p.C456*	c.1368C>A p.C456*	0	0
40	M	c.2643+1G>A (splice site)	c.4577A>C p.K1526T	ND	0
42	M	c.3892A>G p.I1298V	c.1878G>A p.M626I	ND	0
45	F	c.997_998delGC p.A333Qfs*25	c.1053+1G>A (splice site)	ND	0
46	M	c.1834C>T p.Q612*	c.4833A>G p.S1611S	0	0
48	M	c.855+1delG (splice site)	c.3036G>A p.W1012*	ND	0
49	M	c.254_256delAGG p.L85_V86delinsT	c.4795T>C p.C1599R	0	0
53	M	c.937+4A>T (splice site)	c.5979dupA p.E1994Rfs*3	0	0
54	M	c.2643+1G>A (splice site)	c.4577A>C p.K1526T	0	0
58	F	c.1663C>T p.R555W	c.1663C>T p.R555W	0	0
68	F	c.757C>T p.R253W	c.865T>C p.S289P	+	0

1 = immunofluorescence

Western Blot and IF[1] Interpretation
0 = absent or nearly absent
+ = reduced by ~75%
++ = reduced by ~50%
ND = not done

Additional file 1b. Genetic testing results for Becker muscular dystrophy cases

Age (years)	Sex (M/F)	*DMD* variants	IF[1] staining result
4	M	c.2380G>A<br>p.E794K	Reduced to absent staining with all antibodies
4	M	deletion exons 17-44	Reduced to absent staining with all antibodies
4	M	c.2330T>C<br>p.L777P	Reduced with all antibodies; absent staining with actin binding domain, exons 7/8 antibody
5	M	deletion exons 19-28	Reduced to absent staining with rod domain antibody
6	M	deletion exons 45-57	Reduced to absent staining with all antibodies
8	M	c.7189C>T<br>p.Q2397*	Predicts in-frame deletion involving exon 50
8	M	c.6290+3076 A>G<br>p.Thr3055Serfs*1	Reduced to absent staining with all antibodies
9	M	IVS48 splice site mutation	Reduced with all antibodies; absent staining with actin binding domain, exons 7/8 antibody
10	M	duplication exons 3-30	Reduced staining with all antibodies
10	M	duplication exons 5-9	Reduced with all antibodies; absent staining with actin binding domain, exons 7/8 antibody
11	M	c.961-5831C>T<br>(splice site)	Reduced with all antibodies; absent staining with actin binding domain, exons 7/8 antibody
11	M	c.76A>C<br>p.N26H	Reduced with all antibodies; absent staining with actin binding domain, exons 7/8 antibody
12	M	c.6438+47818G>T<br>p.Lys2146Valfs*1	Reduced staining with actin binding domain, exons 7/8 antibody
14	M	deletion exons 45-51	Predicts in-frame deletion involving exons 45-50

14	M	stop exon 41	Reduced staining with all antibodies; absent staining with actin binding domain, exons 7/8 antibody
14	M	duplication exons 2-25	Reduced to absent staining with all antibodies
19	M	c.2949+909 C>T<br>p.Ala984Thrfs*33	Reduced staining with carboxy terminus and with actin binding domain, exons 7/8 antibodies
19	M	deletion exons 46-52	Reduced to absent staining with all antibodies
21	M	deletion exons 45-48	Predicts in-frame deletion involving exons 45-49
22	M	c.239C>A<br>p.A80E	Reduced staining with rod domain antibody
23	M	c.1724T>C<br>p.L575P	Reduced staining with actin binding domain, exons 7/8 antibody
25	M	deletion exons 13-44	Predicts a large, in-frame deletion of at least exons 20-43
28	M	c.10753C>T<br>p.Q3585*	Absent staining with carboxy terminus antibody
31	M	c.9G>A<br>p.W3*	Reduced staining with all antibodies; absent staining with actin binding domain, exons 7/8 antibody
31	M	deletion exons 45-48	Predicts in-frame deletion involving exons 45-46
38	M	deletion exons 45-53	Predicts in-frame deletion involving exons 45-50
42	M	None identified	Predicts in-frame deletion involving exon 50
42	M	c.3780_3786+3del10<br>p.L1261Kfs*20	Reduced staining with carboxy terminus antibody
51	M	None identified	Reduced staining with all antibodies
84	M	duplication exons 45-48	Reduced to absent staining with all antibodies

1 = immunofluorescence

Additional file 1c. Genetic testing results for calpainopathy cases

Age (years)	Sex (M/F)	*CAPN3* variants		Western blot result
		Allele 1	Allele 2	
5	F	deletion of entire gene (plus part of GANC)	deletion of entire gene (plus part of GANC)	0
9	M	c.2462C>T<br>p.A821V	None identified	0
13	F	c.1355-1G>C<br>(splice site)	c.2115+5G>C<br>(splice site)	0
13	F	c.2306G>A<br>p.R769Q	c.2306G>A<br>p.R769Q	0
13	F	c.2092C>T<br>p.R698C	c.2243G>A<br>p.R748Q	+
15	M	c.1981delA<br>p.I661*	c.2440-1G>C<br>(splice site)	0
15	M	c.717delT<br>p.Phe239Leufs*14	c.759_761delGAA<br>p.Lys254del	0
15	M	c.643_663del<br>p.S215_G221del	c.1746-20C>G<br>(splice site)	0
17	M	c.83_delinsCTT<br>p.D295L_fs*57	c.1371C>A<br>p.N457K	+
19	F	c.264T>G<br>p.F88L	c.2362_2363delinsTCATCT<br>p.R788Sfs*14	0
22	M	c.759_761delGAA<br>p.K254del	c.1355-1G>C<br>(splice site)	+
24	M	c.1327T>C<br>p.S443P	c.1505T>C<br>p.1502T	+
25	M	c.481G>A<br>p.G161R	c.1746-20C>G<br>(splice site)	0
26	F	c.550delA<br>p.T184Rfs*36	c.550delA<br>p.T184Rfs*36	0
28	F	c.1468C>T<br>p.R490W	c.1063C>T<br>p.R355W	94kd calpain-3 nearly normal; almost no degradation products

29	F	c.2362_2363delinsTCATCT p.R788Sfs*14	c.533T>C p.I178T	0
31	F	c.1063C>T p.R355W	c.181T>C p.F61L	+
32	F	c.245C>T p.P82L	None identified	0
44	F	c. 245C>T p.P82L	c.1468C>T p.R490W	94kd calpain-3 ++; almost no degradation products
46	M	c.2362_2363delinsTCATCT p.R788Sfs*14	None identified	+
48	F	c.550delA p.T184Rfs*36	None identified	0
53	M	dominant het c.643_663del p.S215_G221del	N/A	+
54	M	dominant het c.643_663del p.S215_G221del	N/A	0
56	M	c.1333G>A p.G445R	None identified	+
62	F	dominant het c.643_663del p.S215_G221del	N/A	+
63	M	None identified	None identified	0
64	F	c.1435A>G p.S479G	None identified	+
66	M	c.1194-9A>G (splice site)	None identified	94kd calpain-3 nearly normal; almost no degradation products
68	M	c.500T>C p.F167S	c.1746-20C>G (splice site)	0
71	M	c.1309C>T p.R437C	deletion of exons 17-20	0

Western Blot Interpretation
0 = absent or nearly absent
+ = reduced by ~75%

++ = reduced by ~50%
