# Supplementary material for: The inflammatory pathology of dysferlinopathy is distinct from calpainopathy, Becker muscular dystrophy, and inflammatory myopathies
Source: Acta Neuropathol Commun. 2022 Feb 8;10:17. doi: 10.1186/s40478-022-01320-z (PMC8822795; doi:10.1186/s40478-022-01320-z)
Supplement: Supplementary file 2 — Additional file 2. Digital quantitation of inflammatory cells per cm2 by diagnosis. [file 40478_2022_1320_MOESM2_ESM.pdf]

Additional file 2. Digital quantitation of inflammatory cells per cm^2 by diagnosis

	CD3+ cells	CD4+ cells	CD8+ cells	CD20+ cells	PU.1+ cells
**DYSF**	603.33 (±105.46)	415.61 (±93.78)	169.34 (±46.02)	50.76 (±15.59)	740.12 (±156.47)
**BMD**	439.84 (±66.57)	403.26 (±104.13)	236.26 (±45.74)	41.32 (±20.20)	511.66 (±121.34)
**CAPN3**	435.51 (±87.73)	392.23 (±105.20)	265.96 (±56.53)	25.14 (±6.44)	623.67 (±198.84)
**DM**	3371.35 (±1210.93)*	1986.71 (±928.95)	729.53 (±307.24)*	753.3 (±375.59)*	2301.55 (±1679.79)
**IBM**	4595.73 (±1725.63)***	2263.13 (±521.84)**	2962.31 (±921.22)***	134.45 (±43.63)	857.85 (±223.23)
**Normal Controls**	86.87 (±21.97)	7.72 (±1.91)	15.76 (±3.48)	29.44 (±8.99)	147.04 (±27.3)

All values are reported as mean (±SEM). * $p = 0.05$. ** $p = 0.01$. *** $p<0.0001$.

DYSF = dysferlinopathy; BMD = Becker muscular dystrophy; CAPN3 = calpainopathy;
DM = dermatomyositis; IBM = inclusion body myositis
